# *Heterometrus spinifer*: An Untapped Source of Anti-Tumor Molecules

**DOI:** 10.3390/biology9070150

**Published:** 2020-07-02

**Authors:** Morhanavallee Soopramanien, Naveed Ahmed Khan, Ajnish Ghimire, Kuppusamy Sagathevan, Ruqaiyyah Siddiqui

**Affiliations:** 1 Department of Biological Sciences, Sunway University, Bandar Sunway, Selangor Darul Ehsan 47500, Malaysia; morhana07@gmail.com (M.S.); ajughi@gmail.com (A.G.); saga@hotmail.com (K.S.); 2 Department of Biology, Chemistry and Environmental Sciences, College of Arts and Sciences, American University of Sharjah, University City, Sharjah 2141, UAE; rsiddiqui@aus.edu

**Keywords:** scorpion, *Heterometrus spinifer*, gut microbiota, anticancer

## Abstract

Despite intensive research, cancer incidence and mortality continue to rise. Consequently, the necessity to develop effective anti-cancer therapy is apparent. We have recently shown that the gut bacteria of animals living in polluted environments, such as crocodiles, are a potential source of novel anti-tumor molecules. To extend this work to other resilient species, we investigated the anti-tumor effects of gut bacteria of *Heterometrus spinifer* (a scorpion). Bacteria from the feces and gut were isolated, identified and evaluated for their anti-tumor effects. Bacterial-conditioned media was prepared in Roswell Park Memorial Institute (RPMI) 1640 media, and cytotoxicity and growth inhibitory properties were examined against cervical (HeLa) cancer cells. Liquid chromatography–mass spectrometry (LC-MS) was conducted to establish the identity of the molecules. Eighteen bacteria species from the gut (HSG01-18) and ten bacteria species from feces (HSF01-10) were tested for anti-tumor effects. Bacterial-conditioned media from scorpion gut and feces exhibited significant growth inhibitory effects against HeLa cells of 66.9% and 83.8%, respectively. Microscopic analysis of cancer cells treated with conditioned media HSG12 and HSG16 revealed apoptosis-like effects. HSG12 was identified as *Pseudomonas aeruginosa* and HSG16 was identified as *Bacillus subtilis*. Both conditioned media exhibited 100% growth inhibitory effects versus a selection of cancer cells, comprising cervical, breast and prostate cancer cells. LC–MS indicated the presence of 72 and 38 compounds, detected from HSG12 and HSG16, respectively. Out of these compounds, 47 were successfully identified while the remainder were unidentified and are possibly novel. This study suggests that the fecal and gut microbiota of scorpions might possess molecules with anti-cancer properties, however, further intensive research is needed to assess these expectations.

## 1. Introduction

The World Health Organization (WHO) reports that most global deaths occur because of noncommunicable diseases; among these cancer incidence and mortality are significantly increasing worldwide, with more than 18 million new cancer cases and 9.6 million cancer deaths in 2018 [1]. The most common causes of death recorded in 2015 were due to lung, stomach, breast, liver and colorectal cancer [2]. Some environmentally induced risk factors include exposure to UV radiation, chemicals, free radicals, sunlight, or other carcinogens [3,4]. The ultimate consequence of carcinogenesis is when healthy cells turn into abnormally growing cells resulting in neoplasm [5]. Additionally, cancer drug resistance presents a significant challenge in the successful prognosis of this debilitating disease [6,7]. Hence, the search for alternative medical treatments for cancer is still ongoing and various natural products are being tested as potential therapeutics [7].

Notably, animals living in polluted environments have existed before humans and often thrive in unhygienic conditions [8]. Despite having exposure to heavy metals and feeding on contaminated foods, animals like cockroaches, crocodiles, scorpions, whales, rats, and water monitor lizards thrive in environments that are detrimental to human health [9], suggesting the presence of potent molecules and/or mechanisms to counter the effects of potential carcinogens. For example, our recent studies have shown that the organ lysates of crocodile (*Crocodylus palustris*) show potent cytotoxicity against prostate (PC3) cancer cells. The findings showed the active compounds present in the organ lysates of the crocodile exhibit anti-tumor properties. The serum and the lysates of the heart induced 70% cell death. This research is significant and could lead to discovery of anti-tumor compounds [10]. The potent anticancer effects in these animals could be due to their immune system. The immune system is a complex network of innate and adaptive components that has the ability to respond to microbial and environmental challenges [11]. Likewise, the gut microbiota has been found to play a fundamental role in the immune system. The gut microbiota produces molecules that can support the immune system to overcome noxious agents [12,13].

In this study, we tested the microbiota of the gut and the feces of the scorpion *Heterometrus spinifer*, a hardy species found in the Malaysian forest. It is shown that the resilient scorpion clade has existed on Earth for at least 450 million years and has been able to survive through various catastrophic events by adapting in physiology and morphology to changes in the environment [14]. Scorpions survive in unhygienic conditions and thrive on germ-infested organisms like crickets, grasshoppers, and mice [15]. Thus it is logical to speculate that they possess mechanisms/molecules to counter harsh/carcinogenic conditions. This is not surprising as other animals including reptiles such as crocodiles, snakes, tortoises and water monitor lizards, live in unsanitary conditions, feed on rotten meat, are exposed to heavy metals such as arsenic, cadmium, cobalt, chromium, mercury, nickel, lead, selenium, endure high levels of radiation, and are among the very few clades to survive the catastrophic Cretaceous-Tertiary extinction event, yet have a prolonged lifespan with minimal cancer incidence [7,8,9,10,11]. A recent study showed the role of metabolomics in mussels in order to assess the effects of environmental pollution [16]. Moreover, some authors have highlighted the effect of anthropogenic loads on the hemocyte population variation in mussels [17]. The overall aim of this study was to determine whether bacteria isolated from scorpions produce molecule(s) that can inhibit growth of cancer cells. A long-term plan is that, if a promising molecule is isolated, it can be synthesized for further testing in vivo as well as clinical testing.

For the first time, we isolated bacteria from the scorpion gut and feces and determined their anti-tumor effects. Cytotoxicity assays and growth inhibition assays were conducted using Henrietta Lacks (HeLa) cervical cancer cells, followed by identification of the molecules with potential anticancer activity using liquid chromatography–mass spectrometry (LC–MS). This is the first comprehensive study investigating the occurrence of anti-tumor molecules and peptides from scorpion gut and feces. However, further studies are essential to confirm the mechanism of the active conditioned media found in this study, such as flow cytometry. Recent studies have shown a new analysis technique important in recognizing cells morphology using flow cytometry [18].

## 2. Materials and Methods

### 2.1. Ethics Committee Consent and Use of Scorpions

The use of scorpion material obtained for this study was granted by Sunway Research Ethics Committee (Research Ethics Approval Code: PGSUNREC 2019/023). Handling of the animal, anesthesia and dissection of the internal organs were all performed by qualified zoologist, Dr K. Sagathevan, who routinely performs such procedures. The scorpion procured for this study was healthy as evident form the absence of lesions and active movement upon stimulation.

### 2.2. Fecal Sample Collection

*Heterometrus spinifer* (scorpion) was procured from the wild, fecal material was collected and inoculated on blood agar and nutrient agar plates using cotton swabs. The agar plates were then incubated overnight at 37 °C, followed by bacteria identification and conditioned media preparation.

### 2.3. Dissection

**Figure 1:**
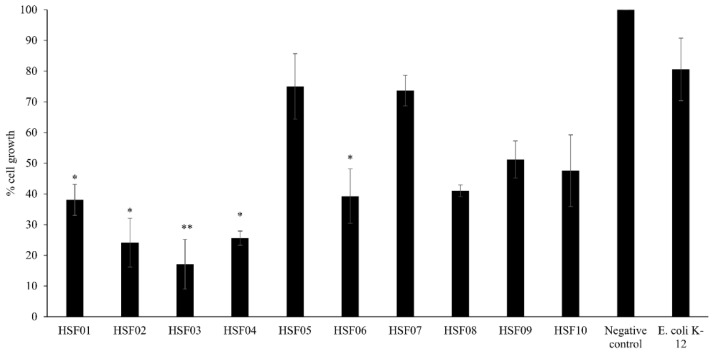
The growth inhibition effect of conditioned media prepared from bacteria isolated from the fecal samples of the scorpion *H. spinifer*. The results represent mean ± standard error of three sets of experiments performed in duplicate for HeLa cells; *p* value was determined using two sample T-test, two-tailed distribution, (*) is <0.05 and (**) is <0.005.

For the dissection of the specimen, the scorpion was immobilized by placing it on ice for 5 min, followed by dissection. The appendages of the scorpion were then removed, followed by the removal of the head. The lower section of the body was then segmented vertically along the midline of the abdomen, exposing the gut (Figure 1). The gut was carefully removed and was then opened with a longitudinal incision. The gut microbes were then isolated from the gut using sterile cotton swabs and dispersed onto nutrient agar and blood agar plates. The agar plates were then kept overnight at 37 °C [13,19].

### 2.4. Bacterial Identification

After overnight incubation, the agar plates were observed, and various bacterial colonies were isolated based on their texture, size, color and shape and were inoculated separately onto fresh blood and nutrient agars. The freshly inoculated plates were kept overnight at 37 °C. Colonies were then evaluated and identified through the analytical profile index (API) [13,19].

### 2.5. Conditioned Medium Preparation

As described previously [13,19], conditioned media were prepared by inoculating single colonies of bacteria isolated in Roswell Park Memorial Institute (RPMI)-1640 medium, followed by 24 h incubation at 37 °C in an aerobic environment with shaking. The cultures were subjected to centrifugation after incubation at 4 °C, 10,000 × *g*, for 1 h. The supernatants for each culture were collected and filtered using a 0.22-μm pore size cellulose acetate syringe filter. The conditioned media were then stored at −80 °C [13,19]. Bradford assay was conducted, using bovine serum albumin (BSA) protein standards and Bradford reagent, to estimate the protein concentration of the conditioned media.

### 2.6. Culture of Cancer and Normal Cells

Prior to anticancer assessment of conditioned media, cancer and normal cells were maintained. For the present study, HeLa (cervical), MCF7 (breast), PC3 (prostate) cells were used, as cervical, breast, and prostate cancer represents a significant burden on human health. Cervical cancer (HeLa (ATCC^®^ CCL2™)), breast cancer (MCF7 (ATCC^®^ HTB-22™)) and prostate cancer (PC3 (ATCC^®^ CRL1435™)) were obtained from the American Type Culture Collection and the normal cell line; Human keratinocyte skin cells (Hacat) (CVCL_0038, CLS:300493), procured from Cell Lines Service (CLS), Germany) were cultivated in RPMI-1640 augmented with 10% fetal bovine serum (FBS), 1% L-glutamine, 1% penicillin streptomycin antibiotic and 1% minimum essential media (MEM) non-essential amino acid at 37 °C, with a supply of 5% carbon dioxide and 95% humidity [10,19].

### 2.7. Growth Inhibition Assays

To determine the growth inhibitory effects of the conditioned media on HeLa cells as stated prior [19], semi-confluent (50% confluency) cells were grown in 96-well plates for 24 h at 37 °C in 5% CO_2_ with 95% humidity. Control wells were selected from the plates and the cells were subjected to Trypan blue exclusion assay, in order to determine the cell count and set to 50% confluency. The cells were treated with the conditioned media prepared from the bacteria isolated from the feces and gut of the animal and conditioned media from *Escherichia coli* K-12 (as a negative control), in a ratio of 1:1 of conditioned media to supplemented RPMI media. The cells were incubated in the same condition; 37 °C in 5% CO_2_ with 95% humidity, until the untreated cells became 100% confluent. The treated and untreated cells were then trypsinized with 2.5% trypsin for 15 min and subjected to Trypan blue exclusion assay using a haemocytometer. The growth inhibition effects were established by comparing the number of viable cells of the untreated cells (control) and treated cells.

### 2.8. Cytotoxicity Assays, Cell Staining and Survival Assays

In order to assess the cytotoxic effects of the conditioned media towards mammalian cells, lactate dehydrogenase (LDH) assay was conducted as described previously [10,19]. For this assay, confluent monolayers of HeLa cancer cells were grown in 96-well plates for 24 h at 37 °C in 5% CO_2_ with 95% humidity. However, in the first part of the study only HeLa cells were selected and treated with conditioned media (CM). HeLa were selected for bioassay-testing as they are representative of commonly observed cervical cancer and because they are fast growing. For the remaining part of the study, once potent CM were identified, the remaining cancer cell lines were utilized for toxicity assays as described. The cells were treated with the conditioned media prepared from the bacteria isolated from the feces and gut of the scorpion and conditioned media from *Escherichia coli* K-12 (as a negative control), for 24 h at 37 °C in 5% CO_2_ with 95% humidity. Following the incubation, a positive control was prepared by treating control cells with 0.2% Triton X-100 for 30 min at 37 °C, to induce 100% cell death. Triton X-100 is a detergent that causes rupturing of the cells resulting in 100% cell death. The supernatant from wells containing treated and untreated cells were collected and subjected to reaction with Roche LDH kit reagent in a 1:1 ratio for 10 min in the dark. An absorbance reading using a spectrophotometer at a wavelength of 490 nm was then taken. The percentage cytotoxicity was calculated as follows:

% cytotoxicity = ((Absorbance_sample_ − Absorbance_negative control_)/(Absorbance_positive control_ − Absorbance_negative control_)) × 100,

where the negative control comprised cells treated with RPMI-1640 media only, and the positive control contained cells treated with the detergent: triton X-100.

Cell staining was conducted by fixing the cells with 100% acetone and 100% methanol in a 1:1 ratio for 15 min, followed by staining the cells with 0.4% Trypan blue solution for 15 min. To further determine the viability of cells treated with CM, survival assays were performed. Briefly, cells treated with CM were collected and seeded onto new plates containing growth media, and their re-growth were examined using a light microscope over a period of several days [15].

### 2.9. Growth Inhibition Assays of Concentrated Active CM

To assess the effects of active CM quantitatively, active CM were concentrated using a vacuum concentrator by at least 10-fold, and the protein concentration of those CM were assessed using the Bradford assay. The potent growth inhibitory effects of active concentrated CM were assessed as mentioned above against a selection of cancer cells; cervical cancer, breast cancer, prostate cancer and normal cell line; at a concentration of 10 µg/mL [19].

### 2.10. Identification of Molecules(s) Exhibiting Anticancer Activity through Liquid Chromatography–Mass Spectrometry (LC–MS)

The samples were prepared by extracting molecules from the conditioned media using 100% chloroform in a 1:3 ratio of chloroform to conditioned media. The chloroform containing the extracted molecules were then subjected to evaporation and the molecules were resuspended in 1:1 ratio of methanol to type 1 water. The samples were scrutinized using Agilent 1290 Infinity LC system coupled to Agilent 6520 Accurate-Mass Q-TOF mass spectrometer with dual electrospray ionization (ESI) source. The molecules were separated using liquid chromatography using Agilent Zorbax Eclipse XDB-C18 column with a particle size of 3.5 micrometers and a narrow-bore of 2.1 × 150 mm at 25 °C using solvent A (0.1% formic acid in water) and solvent B (0.1% formic acid in acetonitrile) for a total run time of 30 min.

The compounds were ionized by means of ESI + jet stream ion mode with the triple-quadrupole (QQQ) analyzer. The parameters of the ion source were established as follows: capillary voltage at 4500 V, sheath gas flow at 8 L per min, fragmentor voltage 135 V, gas temperature at 350 °C, gas flow at 8 L per min, and nebulizer gas at 40 psi. The detector used was an MCP microchannel plate detector, while the blank expended after each sample was of composition 50% MeOH + 50% MilliQ water. The chromatograms produced from mass spectrometry were then used to determine the identity of the molecules from the Metlin_AM_PCDL-N-170502.cdb database. Reported biological activities of the compounds were identified and their novelty were determined with the aid of the SciFinder software (https://sso.cas.org/as/fmIfN/resume/as/authorization.ping).

### 2.11. Statistical Analysis

The data are demonstrative of the mean ± standard error of numerous independent experiments implemented in duplicates. Statistical significance for differences was assessed using a 2-sample t-test; two-tailed distribution, comparing the mean of two different experiments repeated using similar conditions; *p* values of <0.05, <0.01 and <0.005 were employed in the analysis.

## 3. Results

### 3.1. A Spectrum of Bacteria Identified from the Faeces and Gastrointestinal Tract of H. Spinifer

Various bacteria were isolated from the fecal samples and gastrointestinal tract of the Malaysian scorpion and these bacteria were classified as Gram-negative and Gram-positive (Table 1). From the fecal sample, a variety of Gram-positive bacteria were isolated including *Lactobacillus fuchuensis* and *Lysinibacillus fusiformis*; and a panel of Gram-negative bacteria; *Enterobacter gergoviae, Klebsiella pneumoniae, Acinetobacter baumannii, Pseudomonas stutzeri, Enterobacter cloacae* and *Pseudomonas aeruginosa*. From the gastrointestinal tract, Gram-positive, coagulase negative *Staphylococcus* spp., Gram positive *bacilli*, *Staphylococcus sciuri, Bacillus pumilus* and *Bacillus subtilis* and Gram-negative *Pseudomonas aeruginosa, Burkholderia cenocepacia,* and *Serratia marcescens*, were isolated.

### 3.2. Conditioned Media from Feces of H. Spinifer Inhibited the Growth of HeLa Cells

All CM prepared from bacteria isolated from the feces of scorpion exhibited more than 50% growth inhibitory effects against HeLa cells after treatments, except for conditioned media HSF05 and HSF07. However, conditioned media HSF01, HSF02, HSF03, HSF04 and HSF06 prepared from *E. gergoviae*, *K. pneumoniae*, *A. baumannii*, *L. fuchuensis* and *L. fusiformis*, respectively, showed significant growth inhibitory effect towards HeLa cells by reducing the percentage cell growth by 61.9%, 75.9%, 82.9%, 74.4% and 60.7% respectively (Figure 1). The representative microscopic images depict the growth inhibitory effect of the CM prepared from bacteria isolated from the feces of scorpion, except for CM HSF05 and HSF07 towards HeLa cells, as indicated by the confluency and morphology of the cells, in comparison the negative control cells and cells treated with *E. coli* K-12 CM, which showed no effects (data not shown).

### 3.3. Conditioned Media from Gastrointestinal Tract of H. Spinifer Inhibited the Growth of HeLa Cells

**Figure 2:**
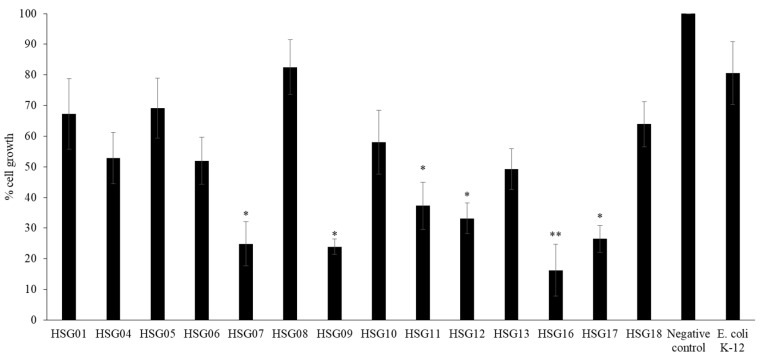
The growth inhibition effect of conditioned media prepared from bacteria isolated from the gastrointestinal tract of scorpion, *H. spinifer* against HeLa cells as described in Materials and Methods. The results represent mean ± standard error of three sets of experiments performed in duplicate for HeLa cells; *p* value was determined using two sample T-test, two-tailed distribution, (*) is < 0.05 and (**) is < 0.005.

Conditioned media prepared from bacteria isolated from the gastrointestinal tract of the scorpion exhibited growth inhibitory effects against HeLa cells. However, only conditioned media HSG07, HSG09, HSG11, HSG12, HSG16, and HSG17 prepared from Gram-positive *bacilli*, *Staphylococcus sciuri*, *Pseudomonas aeruginosa*, *Bacillus subtilis* and *Serratia marcescens*, respectively, inhibited the percentage cell growth by 75.1%, 76.1%, 62.7%, 66.9%, 83.8% and 73.5 % respectively, while HSG01, HSG04, HSG05, HSG06, HSG10 and HSG18 exhibited limited growth inhibitory effects (Figure 2). The representative microscopic images show that HSG07, HSG09, HSG11, HSG12, HSG16, and HSG17 exhibited growth inhibitory effects against HeLa cells, as evident by the distinct morphology of the cells when compared to the negative control cells and cells treated with *E. coli* K-12 CM, which did not show any effects (data not shown).

### 3.4. Conditioned Media from Feces of H. Spinifer Exhibited Limited Cytotoxic Effect Towards HeLa Cells

CM prepared from bacteria isolated from the feces of scorpion exhibited limited cytotoxic effects against HeLa cells as compared to the positive control (cells treated with triton x-100 reagent). The results were supported by representative images of the cells, showing a confluent monolayer of cells similar to untreated cells (negative control). Furthermore, the cell staining experiments indicated that cells were not affected after treatment with the conditioned media as the cells were stained blue, similar to the negative control, indicating that the cells were still viable.

### 3.5. Conditioned Media HSG12 and HSG16 from the Gastrointestinal Tract of H. Spinifer Affected HeLa Cell Morphology

CM from HSG12 and HSG16 resulted in rounding of HeLa cells and a change in morphology as compared to untreated cells (negative control) as depicted in the microscopic images. Nonetheless, the remaining CM displayed confluent monolayers, with similar morphology to the negative control. However, the CM prepared from bacteria isolated from the gastrointestinal tract of scorpion exhibited limited cytotoxic effect against HeLa cells as shown by the percentage cytotoxicity, in comparison to the positive control (cells treated with triton x-100 reagent). Moreover, cell staining experiments revealed that wells containing cells treated with HSG12 and HSG16 remained unstained, as in the positive control; indicating the absence of viable cells, while wells with cells treated with the remaining CM were stained blue, similar to the negative control, indicating that the cells were viable.

### 3.6. HSF02, HSF03, HSF05 and HSF08, from Feces of H. Spinifer, Affected the Survival of HeLa Cells

Post treatment with CM from bacteria isolated from feces of scorpion (HSF01, HSF04, HSF06, HSF07, HSF08, HSF09 and HSF10) HeLa cells were still able to grow, indicating the CM from the fecal sample did not inhibit HeLa cell survival. In contrast, growth of cells treated with HSF02, HSF03, HSF05 and HSF08 was lower, as indicated by cell confluency in comparison with negative control cells, indicating that the HeLa cells were affected after treatment with the respective CM.

### 3.7. HSG12, HSG16 and HSG17 from Gastrointestinal Tract of H. Spinifer, Affected the Survival of HeLa Cells

Following post treatment with CM from bacteria isolated from feces of scorpion, HeLa cells were still able to grow with a similar morphology to the untreated cells and cells treated with CM from *E. coli* K-12, except for cells treated with HSG12 and HSG 16. This indicated the CM from fecal samples (except for HSG12 and HSG16) did not inhibit HeLa cell survival. In contrast, the cells treated with CM HSG12 and HSG16 exhibited a different morphology compared to the negative controls; this indicated that the HeLa cells were affected post treatment with these CM. The results indicated that the growth of cells treated with HSG01, HSG06, and HSG17 was lower, as indicated by the cell confluency compared to the negative control cells and cells treated with CM from *E. coli* K-12.

### 3.8. Concentrated CM from the Gastrointestinal Tract of H. Spinifer Inhibited the Growth of Cancer and Normal Cells

To further investigate the anti-tumor effects of selected CM, the conditioned media were concentrated and subjected to a panel of cancer cells: cervical cancer (HeLa (ATCC^®^ CCL2™)), breast cancer (MCF7 (ATCC^®^ HTB-22™)) and prostate cancer (PC3 (ATCC^®^ CRL1435™)) and normal cell line; and aneuploid immortal keratinocyte (HaCaT), to assess their potential anticancer activity at a concentration of 10 µg/mL, maintaining the same methods as described in the methodology section.

**Figure 3:**
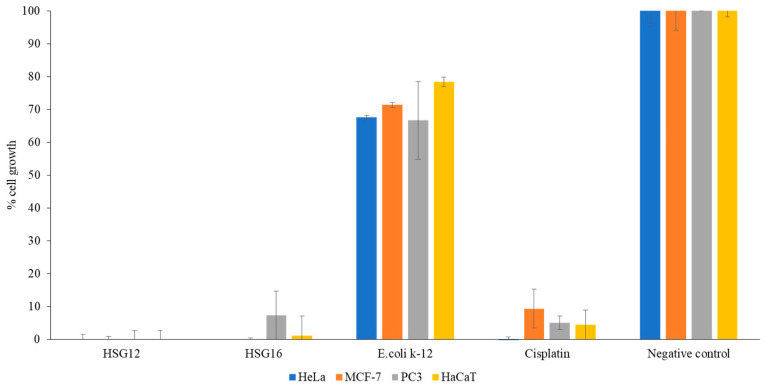
The growth inhibition effect of effective conditioned media (10 µg/mL) prepared from bacteria isolated from the gastrointestinal tract sample of scorpion, *H. spinifer*, against HeLa cells as described in the Materials and Methods. The results represent mean ± standard error of two sets of experiments performed in duplicate for HeLa cells.

CM HSG12 and HSG16 prepared from *P. aeruginosa* and *B. subtilis*, respectively, isolated from the gastrointestinal tract of the scorpion species, exhibited 100% growth inhibitory effect toward cancer and normal cells at a concentration of 10 µg/mL (Figure 3). The quantitative data were further supported by the microscopic images obtained post treatment of the normal and cancer cells. The treated cells did not share the same morphology as the negative control, comprising untreated cells (data not shown).

### 3.9. Concentrated CM from the Gastrointestinal Tract of H. Spinifer Possesses 72 and 38 Molecules

**Figure 4:**
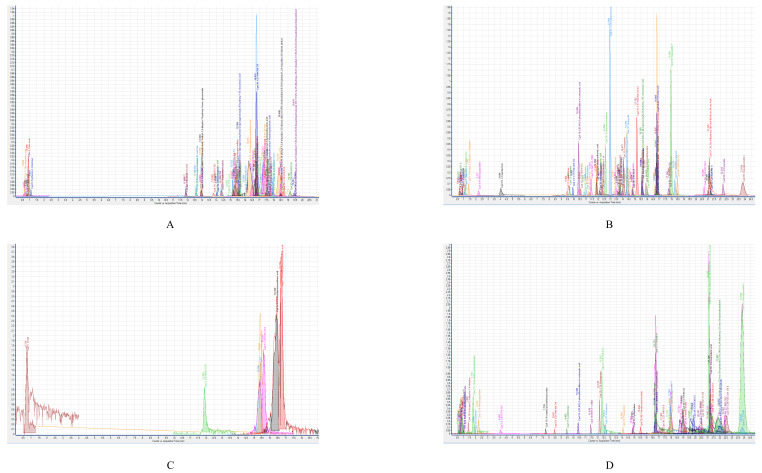
The LC–MS spectra of the effective conditioned media prepared from bacteria isolated from the gastrointestinal tract of scorpion, *H. spinifer*: HSG12 (*P. aeruginosa*) and HSG16 (*B. subtilis*) when subjected to Agilent Technologies 6520 Accurate-Mass Q-TOF mass spectrometer with dual ESI source LC–MS analysis. Briefly the conditioned media was subjected to chloroform extraction and the extract was dried under pressure and dissolved in HPLC-grade methanol for LC–MS analysis. (**A**) The spectrum of molecules detected for negative ion polarity from HSG12. (**B**) The spectrum of molecules detected for positive ion polarity from HSG12. (**C**) The spectrum of molecules detected for negative ion polarity from HSG16. (**D**) The spectrum of molecules detected for positive ion polarity from HSG16.

CM HSG12 and HSG16 prepared from *P. aeruginosa* and *B. subtilis* were subjected to LC–MS (Agilent Technologies 6520 Accurate-Mass Q-TOF mass spectrometer with dual ESI source) to identify the different metabolites synthesized and secreted by the respective bacteria isolated from the gastrointestinal tract of the selected scorpion. Figure 4 shows the spectra (negative and positive ion polarity) of molecules detected from HSG12. A total of 77 molecules were detected from HSG12, out of which 31 molecules were identified (Table 2 and Table S1), while the remaining molecules remained unidentified. Moreover, out of the 32 identified molecules, only 12 had reported biological activity (Table S1) and two had reported anticancer effects; namely U-0126 and proglumide. However, for the remaining 46 unidentified molecules from HSG12, limited information was available: retention time, molecular mass and molecular formula (Table S2).

Figure 4 shows the spectra (negative and positive ion polarity) of molecules detected from HSG16. A total of 38 molecules were detected from HSG16, out of which 15 molecules were identified (Table 2 and Table S1), while the remaining molecules remained unidentified. Moreover, out of the 15 identified molecules, only 7 had reported biological activity (Table S1) and 1 had reported anticancer effects; namely 3-butylidene-7-hydroxyphthalide. However, for the remaining 23 unidentified molecules from HSG16, limited information was available, consisting of retention time, molecular mass and molecular formula (Table S2).

## 4. Discussion

As one of the leading causes of morbidity and mortality, rigorous attempts have been made to find new and effective anti-cancer therapies. In this research, for the first time, the CM from the fecal and gut bacteria of *H. spinifer* were evaluated against cervical cancer cells. Gut bacteria of *H. spinifer* were identified and nine different types of bacteria were investigated further. Coagulase-negative *Staphylococcus* spp., *B. pumilus*, *P. aeruginosa*, Gram-positive *S. sciuri, B. cenocepacia, E. gergoviae, B. subtilis, Serratia marcescens* were identified. Similarly, fecal bacteria of *H. spinifer* identified eight different types of bacteria. Common bacteria found in both gut and feces were *P. aeruginosa* and *E. gergoviae.* Previous studies showed similar findings from the gut bacteria of other species of scorpion [20]. Our results revealed that CM from HSG12 and HSG16 exhibited apoptosis-like effects, such as rounding of cells, and shrinking of cells. HSG12 was identified as *P. aeruginosa* and HSG16 was identified as *B. subtilis*. Furthermore, results from the growth inhibition assay showed that both CM exhibited potent growth inhibition against HeLa cells. Moreover, after concentration, HSG12 and HSG16 exhibited 100% growth inhibitory effects against both cancer (HeLa, MCF-7 and PC3) and normal (HaCaT) cells. These results are corroborated by previous studies, where it was revealed that *P. aeruginosa* induced apoptosis in pancreatic cell lines in a dose-dependent manner, and that the cell cycle progression at S-phase was blocked [21]. In another study, iturin A-like lipopeptides were obtained from *B. subtilis* that induced apoptosis in colorectal adenocarcinoma cells (Caco-2 cells) [22]. Among other factors, bacteria produce a lipopolysaccharide (LPS) endotoxin, that triggers the activation of receptors like Toll-like receptor 4, which inhibits the growth of epithelial tumor cells [23,24].

Our findings are broadly comparable to those of Arimochi et al. [25], who showed that 10% CM prepared from *Clostridium perfringens* resulted in decreased colorectal cancer cell proliferation by 60%, while CM is known to inhibit the cell proliferation as result of breakdown of extracellular matrix (ECM) [26]. In support, in-vivo studies on colorectal cancer cells found that *Staphylococcus aureus* induces response that delays the growth of tumor. Other studies [27] found that the blood extract from crocodiles killed lung cancer cell line A549 in a reactive oxygen species (ROS)-dependent manner as well as increased activities of caspase-3 and caspase-7. Increase in ROS causes disturbance in the mitochondrial membrane potential, possibly resulting into apoptosis. Later studies showed that teixobactin, a compound produced by *Eleftheria terrae*, inhibits cell wall peptidoglycan synthesis [28]. Collectively, these findings suggest that the bacterial strains produce molecules that induce damage to cancer cells.

Results from LC–MS analysis further augmented our findings, as it was revealed that CM prepared from HSG12 and HSG16 of the scorpion’s gastrointestinal tract comprise molecules with previously reported anticancer activity. Namely, U-0126 and proglumide detected from HSG12 and 3-Butylidene-7-hydroxyphthalide detected from HSG16 had reported anticancer activities. U-0126 is an inhibitor of MEK1 and MEK2 kinases that are essential for the activation of growth factors, hormones and cytokines essential for cellular growth, proliferation and differentiation [29,30]. Previous studies corroborate our findings, and it was found that U-0126 inhibited rhabdomyosarcoma tumor growth in embryonal rhabdomyosarcoma (ERMS) cell line-xenotransplanted mice [31]. In another study, it was reported that U-0126 had antiproliferative effects against human endometrial cancer cells (Ishikawa, derived from well-differentiated endometrial cancer; HEC-1A, derived from moderately differentiated endometrial cancer; and AN3CA (obtained from ATCC), derived from undifferentiated endometrial cancer) in a dose-dependent manner, both independently and in combination with COX-2 inhibitor NS398 [32].

On the contrary, proglumide is a drug employed to treat gastrointestinal conditions, which reduces gastric secretions. It was reported that the drug inhibited proliferation and induced apoptosis in gastric cells of the MKN-45 cell line [33]. Furthermore, in another study, it was reported that proglumide exhibited growth inhibitory effects against six human colon cancer cell lines: HCT 116, RKO, MOSER, JVC, FET, and CBS [34]. 3-butylidene-7-hydroxyphthalide identified from conditioned media HSG16 has previously reported anticancer activity; it exhibited cytotoxicity against breast cancer MCF-7 cells [35]. While these identified molecules had reported anticancer activity, the other detected molecules had a broad range of reported biological activities: citric acid, dextromethorphan and 3-butylidene-7-hydroxyphthalide had reported antibacterial activity [36,37,38,39,40]. U-0126, 3-hydroxymorphinan and bifemelane exhibited neuroprotective effects [41,42,43,44,45]. U-0126 had previously reported antiviral activity [46,47]. Moreover, dextromethorphan had reported anti-inflammatory activity and antitussive activity [48,49]. Ferimzone had reported antifungal activity [50].

Nonetheless, the remaining molecules identified from both HSG12 and HSG16 did not have any previously reported biological activity, thus the potential anticancer activity of those molecules needs to be assessed individually and in synergy with other molecules. All in all, we were able to identify a handful of molecules with potential biological activity. The majority of molecules remain unidentified and they are potentially novel. Future studies will involve identification of each molecule, characterization, isolation/synthesis in sufficient quantity, and bioassay testing. However, this study highlight the potential of animals living in unique environments as a source of potentially novel molecules. Future work is needed to identify and assess each molecule, and if proven novel and active, then it could be developed further. In addition, further in-vitro and in-vivo studies are needed using purified compounds. The unidentified molecules detected from the CM with effective activities should be further characterized as they may also be potential anticancer molecules. The 40 and 23 molecules unidentified from CM HSG12 and HSG16 respectively (Table S2) could be prospective candidates with anti-cancer effects. Furthermore, only easily culturable aerobic bacteria were isolated in this study, while anaerobic and unculturable bacteria were not identified from the fecal and gut microbiota of the scorpion. Studying the effect of anaerobic and unculturable bacteria could lead to the discovery of other potential anticancer molecules in the gut microbiota of animals.

## 5. Conclusions

These results of our study show that feces and gut microbiota from the Malaysian scorpion, *H. spinifer*, comprise numerous molecules that may have possible anti-tumor effects. To our knowledge, this is the first time that such an analysis has been carried out, and where the microbiota of these species have been sequestered and their anti-tumor effects identified. LC—MS findings enumerated several molecules that could be possible drug leads, but additional research is needed to accomplish these expectations. It will be imperative to examine the anticancer effects of these microbiota against additional cancer cells types, as well as in vivo. Moreover, as these molecules also affected the normal cells tested, it will be crucial to foster targeted therapy in forthcoming studies, using either nanotechnology or other carriers such as liposomes, so that only cancer cells are targeted. This work further supports our hypothesis that animals residing in unsanitary habitats that are often unfavorable to humans, are a large untapped resource for anticancer drugs.

## Figures and Tables

**Table 1:** Bacterial species isolated from the feces and gastrointestinal tract of the scorpion *H. spinifer*.

	Gram Stain	Bacteria
**Scorpion faecal samples**		
HSF01	Gram negative	*Enterobacter gergoviae*
HSF02	Gram negative	*Klebsiella pneumoniae*
HSF03	Gram-negative	*Acinetobacter baumannii*
HSF04	Gram-positive	*Lactobacillus fuchuensis*
HSF05	Gram-negative	*Pseudomonas aeruginosa*
HSF06	Gram-positive	*Lysinibacillus fusiformis*
HSF07	Gram-negative	*Pseudomonas aeruginosa*
HSF08	Gram-negative	*Pseudomonas stutzeri*
HSF09	Gram-negative	*Pseudomonas aeruginosa*
HSF10	Gram-negative	*Enterobacter cloacae*
**Scorpion gut**		
HSG01	Gram-positive	Coagulase negative *Staphylococcus* spp.
HSG04	Gram-positive	Coagulase negative *Staphylococcus* spp.
HSG05	Gram-positive	*Bacillus pumilus*
HSG06	Gram-negative	*Pseudomonas aeruginosa*
HSG07	Gram-positive	Gram-positive *bacilli*
HSG08	Gram-positive	Coagulase negative *Staphylococcus* spp.
HSG09	Gram-positive	*Staphylococcus sciuri*
HSG10	Gram-negative	*Burkholderia cenocepacia*
HSG11	Gram-negative	*Pseudomonas aeruginosa*
HSG12	Gram-negative	*Pseudomonas aeruginosa*
HSG13	Gram-negative	*Enterobacter gergoviae*
HSG16	Gram-positive	*Bacillus subtilis*
HSG17	Gram-negative	*Serratia marcescens*
HSG18	Gram-positive	Coagulase negative *Staphylococcus* spp.

**Table 2:** The molecules detected, identified and unidentified from the conditioned media prepared from bacteria isolated from the gastrointestinal tract of *H. spinifer* through liquid chromatography–mass spectrometry (LC–MS). The conditioned media prepared were subjected to chloroform extraction and the extracts were subject to LC–MS analysis. The spectra generated were searched in the METLIN library in order to reveal the potential identity of the detected molecules. To assess whether those identified molecules had previously reported biological activity, they were then searched in the SciFinder database.

Bacteria	Number of Molecules				
	Detected	Identified	Reported Activity	Anticancer Activity	Unidentified
***H. spinifer***					
*P. aeruginosa* (HSG12)	72	32	11 (Table S1A)	2 U-0126, Proglumide	40 (Table S1B)
*B. subtilis* (HSG16)	38	15	6 (Table S2A)	1 3-Butylidene-7-hydroxyphthalide	23 (Table S2B)

## Supplementary Materials

The following are available online at https://www.mdpi.com/2079-7737/9/7/150/s1, Table S1: Molecules identified from the conditioned media prepared from (A) HSG12 (*P. aeruginosa*) and (B) HSG16 (*B. subtilis*) isolated from gastrointestinal tract of *H. spinifer* through LC—MS. The conditioned media prepared was subjected to chloroform extraction and the extracts were subject to LC—MS analysis. The spectra generated were searched in the METLIN library in order to reveal the potential identity of the detected molecules. To assess whether those identified molecules had previously reported biological activity, they were searched in the SciFinder database, Table S2: Molecules that remained unidentified from the conditioned media prepared from (A) HSG12 (*P. aeruginosa*) and (B) HSG16 (*B. subtilis*) isolated from gastrointestinal tract of *H. spinifer* through LC—MS. The conditioned media was subjected to chloroform extraction and the extracts were subject to LC—MS analysis. The spectra generated were searched in the METLIN library in order to reveal the potential identity of the detected molecules.
