# Supplementary material for: Heterometrus spinifer: An Untapped Source of Anti-Tumor Molecules
Source: Biology (Basel). 2020 Jul 2;9(7):150. doi: 10.3390/biology9070150 (PMC7408436; doi:10.3390/biology9070150)
Supplement: Supplementary file 1 [file biology-09-00150-s001.pdf]

biology

MDPI

Supplementary Table S1: Molecules identified from the conditioned media prepared from (A) HSG12 (*P. aeruginosa*) and (B) HSG16 (*B. subtilis*) isolated from gastrointestinal tract of *H. spinifer* through LC-MS. The conditioned media prepared was subjected to chloroform extraction and the extracts were subject to LC-MS analysis. The spectre generated were searched in the METLIN library in order to reveal the potential identity of the detected molecules. To assess whether those identified molecules had previously reported biological activity, they were searched in the SciFinder database.

(A)

HSG12 (*P. aeruginosa*) Conditioned Media

	Compound	Mass	*m/z*	Reported Activity
1	**Glucoheptonic acid**<br>Molecular formula: $C_7H_{14}O_8$<br>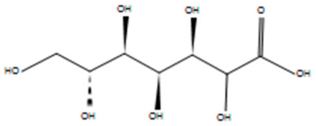	226.07	225.06	No reported biological activity
2	**Citric acid**<br>Molecular formula: $C_6H_8O_7$<br>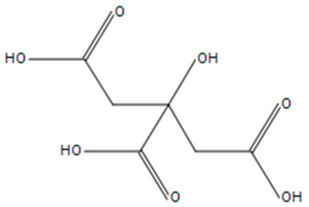	192.03	191.02	*Weak organic acid involved in citric acid cycle during respiration.<br>*Used as flavouring and preservative in food and beverages.<br>*Used a chelating agent.<br>*Antimicrobial activity [37,51].
3	**Erythrono-1,4-lactone**<br>Molecular formula: $C_4H_6O_4$<br>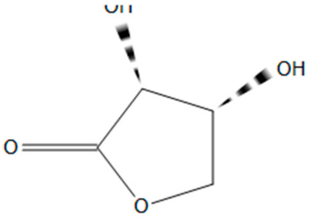	118.03	117.02	*No reported biological activity
4	**U-0126**<br>Molecular formula: $C_{18}H_{16}N_6S_2$<br>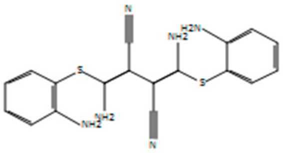	380.09	379.08	*MAPK/ERK kinase activity<br>*Anticancer activity (Antiproliferative effect) [32,33]<br>*Antiviral activity [47,48].<br>*Neuroprotective effect [43,44].
5	**3-Hydroxymorphinan**<br>Molecular formula: $C_{16}H_{21}NO$<br>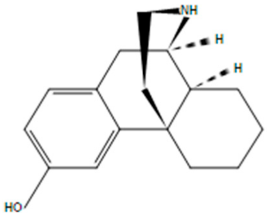	243.16	242.16	*A psychoactive drug of the morphinan family<br>*Neuroprotective agent for the treatment of Parkinson's disease (PD) [42,45].
6	**(10S)-Juvenile hormone III acid diol**<br>Molecular formula: $C_{15}H_{26}O_4$	270.18	305.15	*No reported biological activity

	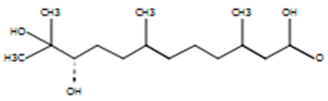			
7	**1,8-Naphthyridine-3-carboxylic acid, 1-ethyl-1,4-dihydro-7-hydroxy-4-oxo- glucuronide**<br>Molecular formula: $C_{17}H_{18}N_2O_{10}$<br>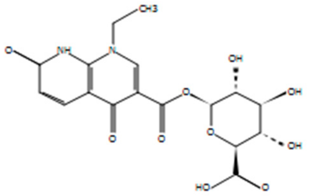	410.10	409.09	*No reported biological activity
8	**3-oxo-tridecanoic acid**<br>Molecular formula: $C_{13}H_{24}O_3$<br>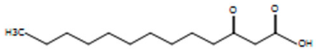	228.17	227.17	*No reported biological activity
9	**Dextromethorphan**<br>Molecular formula: $C_{18}H_{25}NO$<br>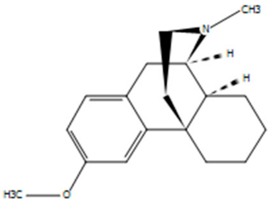	271.19	270.19	* Antitussive activity [49].<br>*Antibacterial activity [38,40,41].<br>*Anti-inflammatory activity [40].
10	**Proglumide**<br>Molecular formula: $C_{18}H_{26}N_2O_4$<br>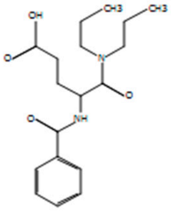	334.19	333.18	*Anticancer activity [34,52].<br>*Acts as gastric antagonist binding to gastrin receptors [53].
11	**2-chloropalmitaldehyde**<br>Molecular formula: $C_{16}H_{31}ClO$<br>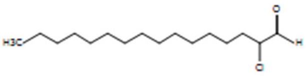	274.21	309.18	*No reported biological activity
12	**3-(2,4-Cyclopentadien-1-ylidene)pregn-4-en-20-one**<br>Molecular formula: $C_{26}H_{34}O$<br>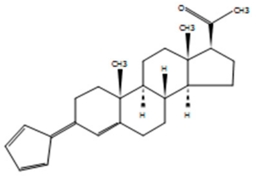	362.26	397.23	*No reported biological activity
13	**(6R)-vitamin D3 6,19-(4-phenyl-1,2,4-triazoline-3,5-dione) adduct / (6R)-cholecalciferol 6,19-(4-phenyl-1,2,4-triazoline-3,5-dione) adduct**<br>Molecular formula: $C_{35}H_{49}N_3O_3$	559.38	594.35	*No reported biological activity

	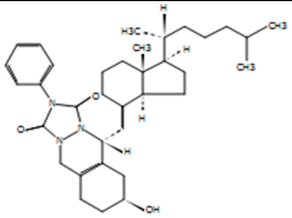			
14	**Trolamine** Molecular formula: $C_6H_{15}NO_3$ 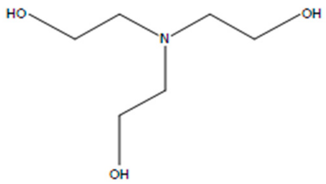	149.11	150.11	*No reported biological activity
15	**Thiodiglycol** Molecular formula: $C_4H_{10}O_2S$ 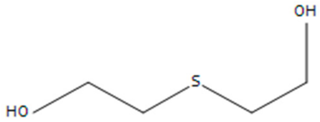	122.04	123.05	*No reported biological activity
16	**1-(1-Pyrrolidinyl)-2-propanone** Molecular formula: $C_7H_{13}NO$ 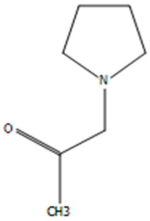	127.10	128.11	*No reported biological activity
17	**CMN 131** Molecular formula: $C_7H_8N_2S$ 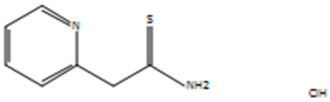	152.04	170.07	*No reported biological activity
18	**5-Acetyl-2,3-dihydro-1,4-thiazine** Molecular formula: C_6H_9NOS 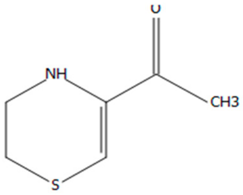	143.04	144.05	*Flavoring agent [54].
19	**(2R,4R)-2-phenylthiazolidine-4-carboxylic acid** Molecular formula: $C_{10}H_{11}NO_2S$ 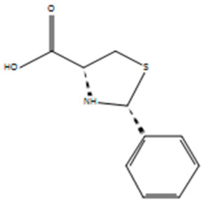	209.05	210.06	*No reported biological activity

20	**Xylylcarb**<br>Molecular formula: $C_{10}H_{13}NO_2$<br>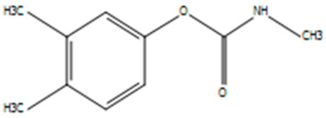	179.09	180.10	*Used in insecticide.
21	**2,6-Nonadien-1-ol**<br>Molecular formula: $C_9H_{16}O$<br>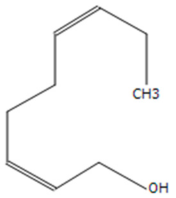	140.12	158.15	*Antiperspirant and deodorant compositions (United States Patent 9730878)
22	**2-Hydroxyethylclavam**<br>Molecular formula: $C_7H_{11}NO_3$<br>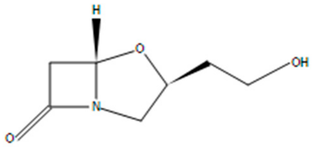	157.07	158.08	*No reported biological activity
23	**Levorphanol**<br>Molecular formula: $C_{17}H_{23}NO$<br>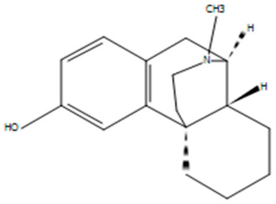	257.18	258.19	* Levorphanol mimics the actions of morphine<br>*Analgesic activity (pain reliever) [55].
24	**D-Vacciniin**<br>Molecular formula: $C_{13}H_{16}O_7$<br>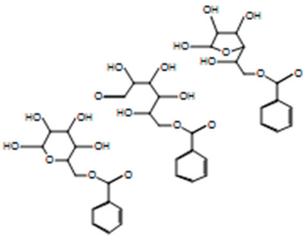	284.09	323.05	*No reported biological activity
25	**Bifemelane**<br>Molecular formula: $C_{18}H_{23}NO$<br>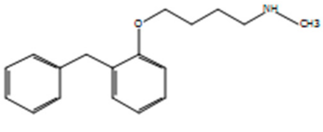	269.18	270.19	*Antidepressant activity [56].<br>*Neuroprotective effect [46].
26	**Prosopinine**<br>Molecular formula: $C_{16}H_{33}NO_3$<br>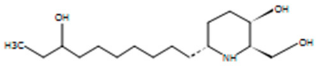	287.25	288.25	*No reported biological activity
27	**Anacyclin**	271.19	272.20	*No reported biological activity

	Compound	Mass	*m/z*	Reported activity
	Molecular formula: $C_{18}H_{25}NO$ 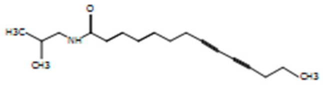			
28	**Spirasine I** Molecular formula: $C_{22}H_{29}NO_3$ 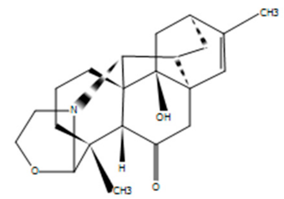	355.22	373.25	*No reported biological activity
29	**Benzphetamine** Molecular formula: $C_{17}H_{21}N$ 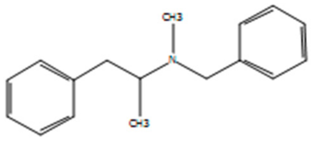	239.17	278.13	*Method of weight management (U.S Patent: 61711413)
30	**Buprofezin** Molecular formula: $C_{16}H_{23}N_3OS$ 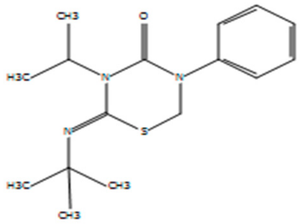	305.16	306.16	*Insecticide [57].
31	**Arachidoyl glycine** Molecular formula: $C_{22}H_{43}NO_3$ 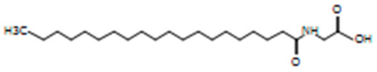	369.32	370.33	*No reported biological activity

(B)

HSG16 (*B. subtilis*) conditioned media

	Compound	Mass	*m/z*	Reported activity
1	**Propionylglycine** Molecular formula: $C_5H_9NO_3$ 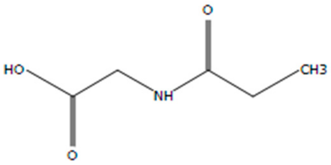	131.06	132.07	*No reported biological activity
2	**6-Aminonicotinamide** Molecular formula: $C_6H_7N_3O$ 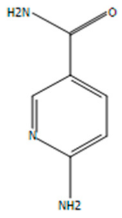	137.06	138.07	*Inhibitor of 6-phosphogluconate (6PG), inhibiting the pentose cycle [58].

3	**Fibrin**<br>Molecular formula: $C_5H_{11}N_3O_2$<br>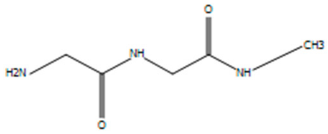	145.08	146.09	* A protein involved in blood clotting [59].
4	**Thiodiglycol**<br>Molecular formula: $C_4H_{10}O_2S$<br>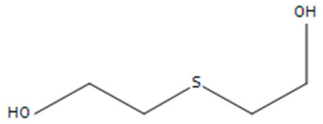	122.04	123.05	*No reported biological activity
5	**Pyroglutamic acid**<br>Molecular formula: $C_5H_7NO_3$<br>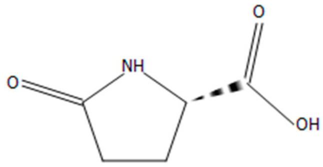	129.04	130.05	*An amino acid, derivative of L-glutamic acid [60].
6	**N-Hydroxy-L-phenylalanine**<br>Molecular formula: $C_9H_{11}NO_3$<br>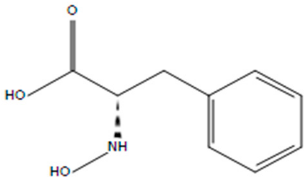	181.07	182.08	*No reported biological activity
7	**N-(2-Methylpropyl)acetamide**<br>Molecular formula: $C_6H_{13}NO$<br>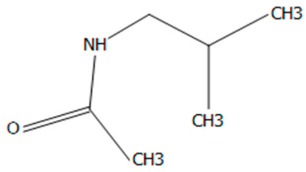	115.10	138.09	*No reported biological activity
8	**5-Acetyl-2,3-dihydro-1,4-thiazine**<br>Molecular formula: C_6H_9NOS<br>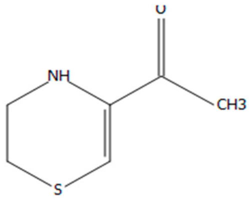	143.04	144.05	*Flavoring agent [54].
9	**Pyrrolidinecarboxaldehyde**<br>Molecular formula: C_5H_9NO<br>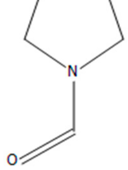	99.07	100.08	*No reported biological activity

10	**N-methyl arachidonoyl amine**<br>Molecular formula: $C_{21}H_{35}NO$<br>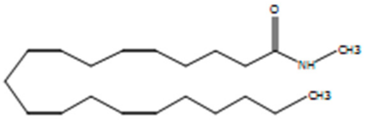	317.27	340.26	*No reported biological activity
11	**(2R,4R)-2-phenylthiazolidine-4-carboxylic acid**<br>Molecular formula: $C_{10}H_{11}NO_2S$<br>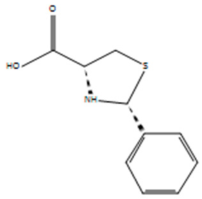	209.05	210.06	*No reported biological activity
12	**Ferimzone**<br>Molecular formula: $C_{15}H_{18}N_4$<br>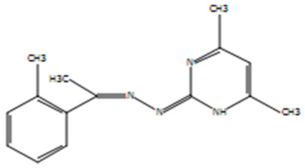	254.15	272.19	*Antifungal activity [50].
13	**Prosopinine**<br>Molecular formula: $C_{16}H_{33}NO_3$<br>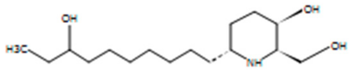	287.25	288.25	*No reported biological activity
14	**Cyclandelate**<br>Molecular formula: $C_{17}H_{24}O_3$<br>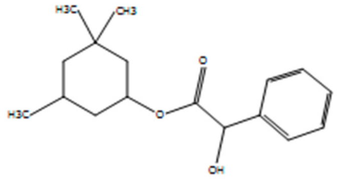	276.17	277.18	*A vasodilator agent [61,62].
15	**3-Butylidene-7-hydroxyphthalide**<br>Molecular formula: $C_{12}H_{12}O_3$<br>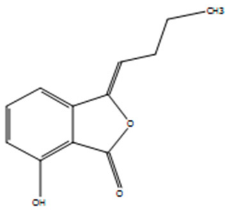	204.08	205.09	*Anti-inflammatory activity [63].<br>*Anticancer activity [64,65].<br>*Antibacterial activity [39].

Supplementary Table S2: Molecules that remained unidentified from the conditioned media prepared from (A) HSG12 (*P. aeruginosa*) and (B) HSG16 (*B. subtilis*) isolated from gastrointestinal tract of *H. spinifer* through LC-MS. The conditioned media was subjected to chloroform extraction and the extracts were subject to LC-MS analysis. The spectre generated were searched in the METLIN library in order to reveal the potential identity of the detected molecules.

**(A)**			
**HSG12 (*P. aeruginosa*) Conditioned Media**			
**Compound**	**Retention Time**	**Molecular Mass**	**Molecular Formula**
1	0.691	147.9731	
2	0.909	112.0167	C5 H4 O3
3	11.877	443.0826	C18 H17 N7 O3 S2
4	12.959	364.0925	C17 H20 N2 O3 S2
5	13.809	459.0597	C18 H25 N3 O S5
6	14.061	333.2529	C18 H31 N5 O
7	15.153	380.0691	C17 H20 N2 O2 S3
8	15.155	443.0656	C18 H17 N7 O S3
9	15.203	476.3001	C25 H40 N4 O5
10	15.302	298.1618	C16 H26 O3 S
11	15.559	713.3832	C32 H59 N O16
12	15.582	566.3428	C27 H46 N6 O7
13	15.586	549.3532	C28 H47 N5 O6
14	15.632	298.161	C16 H26 O3 S
15	15.984	490.3148	C26 H42 N4 O5
16	16.704	312.1777	C17 H28 O3 S
17	16.797	1008.6586	
18	16.801	504.332	C27 H44 N4 O5
19	16.801	1030.64	
20	16.804	567.3277	C27 H45 N5 O8
21	17.007	354.2097	C17 H30 N4 O2 S
22	17.214	678.4203	C35 H58 N4 O9
23	17.323	326.1934	C14 H30 O8
24	17.75	593.342	C29 H47 N5 O8
25	17.752	530.3444	C25 H42 N10 O3
26	17.943	530.2935	C21 H34 N14 O3
27	18.476	532.3619	C28 H52 O9
28	18.657	338.2149	C10 H22 N14
29	19.151	382.2404	C19 H34 N4 O2 S
30	0.673	185.0627	C7 H11 N3 O S
31	0.753	122.0408	C4 H10 O2 S
32	9.931	229.0874	C11 H16 Cl N O2
33	10.564	231.1838	C12 H25 N O3
34	12.335	259.2151	C14 H29 N O3
35	12.964	364.0921	C17 H20 N2 O3 S2
36	13.729	358.0219	C14 H15 Cl N2 O3 S2
37	13.817	396.0644	C17 H20 N2 O3 S3
38	15.162	380.0702	C17 H20 N2 O2 S3
39	15.594	503.3461	C26 H49 N O8
40	16.807	1030.6433	
41	16.809	504.3308	C27 H44 N4 O5
42	17.756	547.3727	C28 H53 N O9
43	18.479	532.3618	C28 H52 O9
44	20.668	397.3508	C23 H47 N3 S
45	20.981	267.1752	C16 H26 Cl N
46	21.198	370.2973	C24 H38 N2 O
**(B)**			

**HSG16 (*B. subtilis*) conditioned media**			
**Compound**	**Retention time**	**Molecular mass**	**Molecular formula**
1	0.739	196.8188	
2	11.902	266.0004	C6 H6 N2 O10
3	15.356	298.1614	C16 H26 O3 S
4	15.631	298.1614	C16 H26 O3 S
5	0.636	119.9592	
6	1.943	143.0929	C3 H9 N7
7	4.048	159.125	C8 H17 N O2
8	8.467	452.3359	C24 H44 N4 O4
9	12.342	157.1465	C9 H19 N O
10	16.74	148.0155	C8 H4 O3
11	17.3	340.2795	C21 H40 O S
12	18.005	274.251	C16 H34 O3
13	18.743	288.266	C17 H36 O3
14	18.968	288.2668	C17 H36 O3
15	19.593	302.281	C18 H38 O3
16	19.85	302.2825	C18 H38 O3
17	20.521	610.1548	C39 H27 Cl O5
18	21.101	148.0157	C8 H4 O3
19	21.104	497.2366	C23 H35 N3 O9
20	21.336	701.207	C44 H32 Cl N3 O4
21	22.381	775.2213	
22	22.562	609.174	C36 H32 Cl N O4 S
23	23.839	762.4992	

© 2020 by the authors. Submitted for possible open access publication under the terms and conditions of the Creative Commons Attribution (CC BY) license (http://creativecommons.org/licenses/by/4.0/).
